# Chemical diversity of dietary phytochemicals and their mode of chemoprevention

**DOI:** 10.1016/j.btre.2021.e00633

**Published:** 2021-05-18

**Authors:** Srimanta Patra, Rabindra Nayak, Suryamani Patro, Biswajita Pradhan, Brundaban Sahu, Chhandashree Behera, Sujit Kumar Bhutia, Mrutyunjay Jena

**Affiliations:** a Cancer and Cell Death Laboratory, Department of Life Science, National Institute of Technology Rourkela, India; b Post Graduate Department of Botany, Berhampur University, Bhanja Bihar, Berhampur, 760007, India; c Department of Home Science, S.B.R. Govt. Women’s College, Berhampur, 760001, India; d College of Fisheries (OUAT), Berhampur, 760007, India

**Keywords:** Apoptosis, Autophagy, Cancer, Chemoprevention, Dietary phytochemical

## Abstract

Despite the advancement in prognosis, diagnosis and treatment, cancer has emerged as the second leading cause of disease-associated death across the globe. With the remarkable application of synthetic drugs in cancer therapy and the onset of therapy-associated adverse effects, dietary phytochemicals have been materialized as potent anti-cancer drugs owing to their antioxidant, apoptosis and autophagy modulating activities. With dynamic regulation of apoptosis and autophagy in association with cell cycle regulation, inhibition in cellular proliferation, invasion and migration, dietary phytochemicals have emerged as potent anti-cancer pharmacophores. Dietary phytochemicals or their synthetic analogous as individual drug candidates or in combination with FDA approved chemotherapeutic drugs have exhibited potent anti-cancer efficacy. With the advancement in cancer therapeutics, dietary phytochemicals hold high prevalence for their use as precision and personalized medicine to replace conventional chemotherapeutic drugs. Hence, keeping these perspectives in mind, this review focuses on the diversity of dietary phytochemicals and their molecular mechanism of action in several cancer subtypes and tumor entities. Understanding the possible molecular key players involved, the use of dietary phytochemicals will thrive a new horizon in cancer therapy.

## Highlights

• Chemically diverse dietary phytochemicals exhibit anticancer activity owing to their antioxidant potential.
• Dietary phytochemicals act as blocking and suppressing agents for exposition of chemoprevention.
• Potent apoptosis and autophagy modulation is chief contributor for exhibition of anticancer efficacy.

## 1 Introduction

Despite the advancement in prognosis, diagnosis and treatment, cancer has emerged as the second leading cause of disease-associated death across the globe [1,2]. According to the reports given by WHO, cancer-associated mortalities have been estimated to be 8.8 million that instituting about 16.66 % of the total death cases as of 2015 [1]. With a primary focus on radiotherapy, chemotherapy, surgery and synergistic drug treatment approaches, cancer treatment has emerged as a high cost regulating approach with several adverse treatment related adverse modalities [3,4]. Moreover, the conventional approaches also have adverse consequences of disease recurrence. With the dire need of abiding by the negative consequences, phytochemicals have emerged as potent non-toxic, chemotherapeutic agents in cancer therapeutics [3,4].

Diet plays an important role in cancer prevention as more than 33 % of the cancer-associated mortalities can be avoided by a change in lifestyle and dietary habits with proper nutritional supplements [[5], [6], [7], [8]]. Dietary phytochemicals, the non-nutritive disease-preventing bioactive compounds constituting polyphenols, carotenoids, glucosinolates, organosulphides, nitrogen-containing compounds and terpenoids are abundantly found in daily dietary supplements have immense potential as chemotherapeutic agents [5,9,10]. These bioactive dietary phytochemicals exert non-toxic and target specific chemotherapeutic action with effective bioactivity and enhanced bioavailability as individual drug candidates or in synergism with conventional chemotherapeutic drugs. Mechanistically, these dietary phytochemicals scavenge reactive oxygen species (ROS) produced in the cellular compartments due to defective cellular metabolism that in turn inhibit cancer progression [5,6,10,11]. With the regulation of several cell death pathways such as apoptosis and autophagy in colossal association with cell cycle regulation, inhibition in cellular proliferation, invasion and migration, dietary phytochemicals exhibit enhanced anticancer activity [[12], [13], [14], [15], [16], [17]].

With more focus on identification and evaluation of anti-cancer efficacy of these dietary phytochemicals, their mode of action, molecular pathways associated with such activity, bioavailability and bioactivity, several studies have been made on the anti-cancer propensity of dietary phytochemicals in numerous cell lines and tumor entities [7,[18], [19], [20]]. Hence with the explosion in cancer incidences globally and the escalating use of dietary phytochemicals, this review has focused on the chemical diversity of dietary phytochemicals and their molecular mode of action in several cancer subtypes.

## 2 Chemical diversity and classification of dietary phytochemicals

**Fig. 1:**
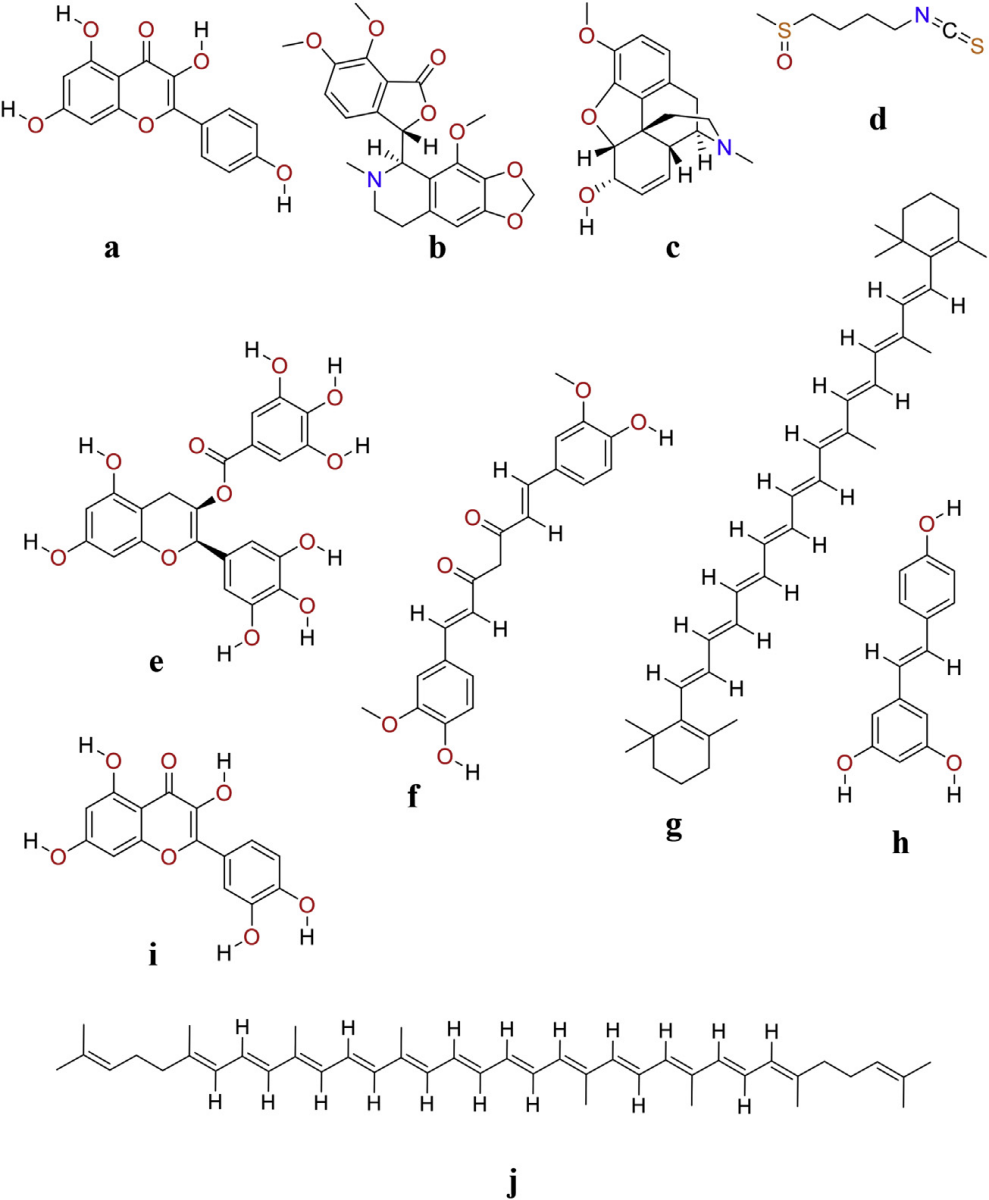
Chemical structure of chemo-preventive dietary phytochemicals. Kaempferol (a), Noscapine (b), Codeine (c), Sulforaphane (d), EGCG (e), Curcumin (f), β-carotene (g), Resveratrol (h), Quercetin (i) and Lycopene (j).

**Fig. 2:**
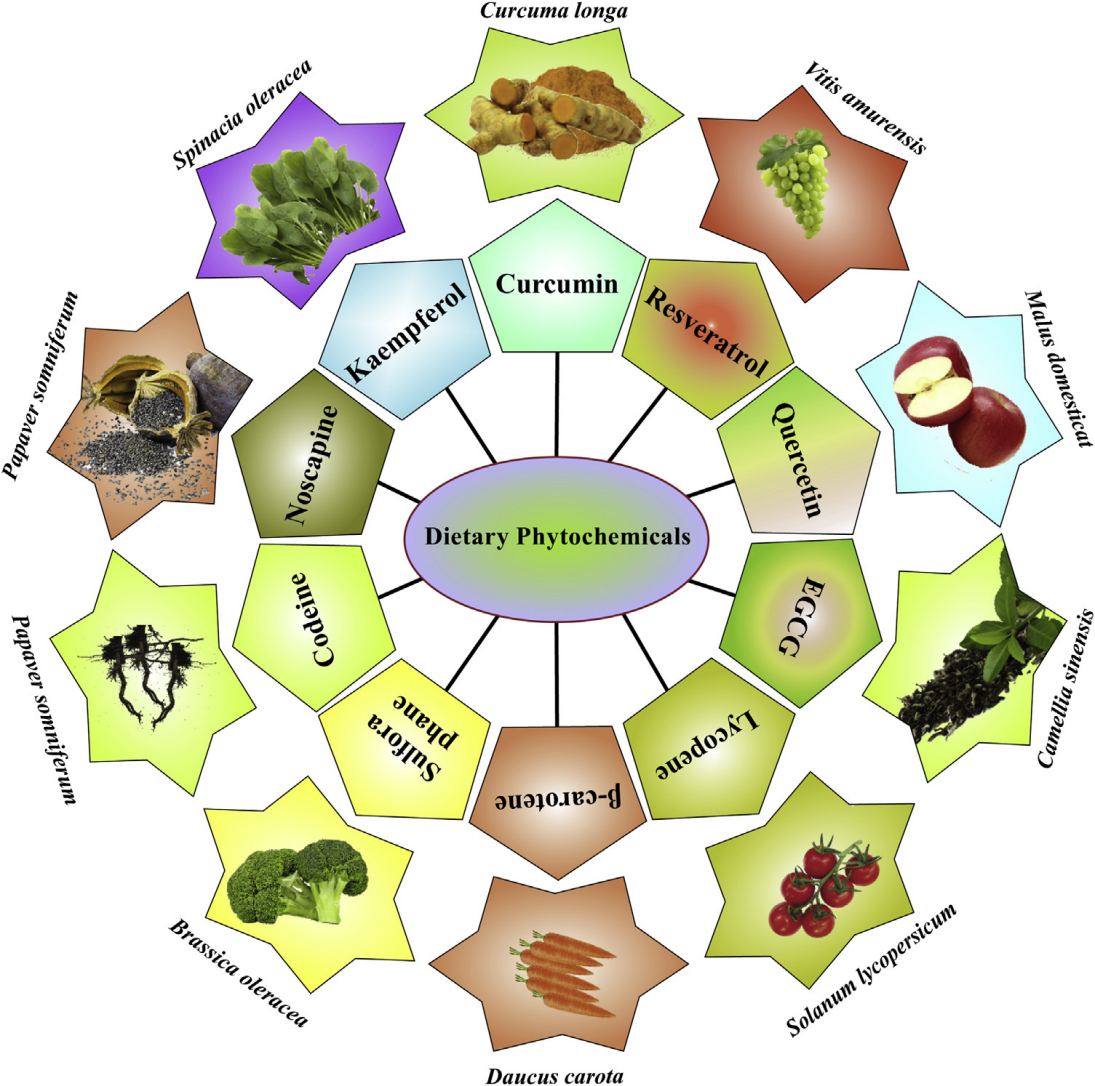
Dietary sources of chemo-preventive dietary phytochemicals. Dietary polyphenols, carotenoids, terpenoids, glucosinolates, organosulphides and nitrogen containing compounds exhibit potent chemo-preventive activity against several cancers. Dietary polyphenols such as curcumin, resveratrol, quercetin, kaempferol, EGCG and carotenoids like β-carotene and lycopene exhibit potent chemo-prevention. In addition to this, glucosinolates and organosulphides such as sulforaphane also demonstrate potent anti-cancer efficacy. Nitrogen containing compounds like codeine and noscapine also display potent anti-cancer efficacy in several cancer cells.

Dietary phytochemicals are non-nutritive disease preventive bioactive plant chemicals that can be used directly as food, food additives or as food adjuvants [[5], [6], [7], [8]]. It has been investigated that over 5000 distinct dietary phytochemicals have been recognized, isolated and screened for their biological activity. The variable biological activity of these dietary phytochemicals against cancer are due to the diverse chemical classes, huge molecular confirmations and structural complexities [[21], [22], [23], [24], [25]]. Moreover, the diverse chemical classifications are also responsible for the enhanced antioxidative and pro-oxidative activity displayed by these dietary phytochemicals. The various class of dietary phytochemicals can be grouped into major divisions of polyphenolics, carotenoids, glucosinolates, organosulfur compounds, nitrogen-containing compounds and terpenoids. An account of various types of dietary phytochemicals has been described below (Fig. 1, Fig. 2, Table 1).

**Table 1:** Overview of chemical diversity of dietary phytochemicals.

Phytochemicals		Types and Example
Group	Sub-Group	
**Polyphenols**	**Flavonoids**	**Flavanols :** Catechin, Gallate & Epigallocatechin
		**Flavonol :** Quercetin, Gingerol & Kaempferol
		**Flavones :** Apigenin & Fisetin
		**Isoflavonoids :** Genistein
		**Anthocyanidin**
	**Non-flavonoids**	**Phenolic acids:** Hydroxybenzoic acid (Rosmarinic acid) & Hydroxycinnamic acids (Curcumin)
		**Stilbenes** : Resveratrol, Lignan
**Carotenoids**	β-Carotene, Lycopene, Crocetin	
**Glucosinolates**	Isothiocyanates, Indole and Sulforaphane	
**Organosulphides**	Allium compounds	
**Nitrogen compounds**	**Alkaloids**	Caffeine, Codeine, Noscopine & Quinidine
	**Capsaicinoids**	Dihydrocapsaicin, Homocapsaicin, Capsaicin, Nonivamide
**Terpenoids**	d-Limonene, d-Carvone, Perillyl alcohol, Andrographolide, Excisanin A, Gnidimacrin, Oridonin, Cucurbitacin B	

### 2.1 Polyphenols

Polyphenols are the major secondary plant metabolites and dietary phytochemicals present in the human diets. Based on the numeral phenol rings and the structural essential elements in the side chain that bind to these phenol rings, polyphenol can be majorly grouped into two subdivisions such as flavonoids and non-flavonoids [5,9,10]. The flavonoids (or bioflavonoids) can be identified as a group of phenolic compounds with a 15-carbon skeleton structure. Flavonoids possess strong antioxidant activity that have a defined role in minimizing the risk of age-related chronic diseases like cancer [5,6,10,11]. The flavonoid group comprises of; flavones (fisetin and apigenin), flavonols (quercetin, kaempferol and gingerol), flavanols (catechin, gallate and epigallocatechin), isoflavonoids (genistein) and anthocyanidin [[26], [27], [28], [29], [30], [31]]. The non-flavonoids comprise phenolic acids and stilbenes. The phenolic acids are subdivided into two chief groups such as; hydroxycinnamic acids and hydroxybenzoic acids. The curcumin is included under hydroxycinnamic acid derivatives whereas rosmarinic acid is grouped under hydroxybenzoic acid derivatives. Resveratrol and lignan are majorly classified under stilbenes.

### 2.2 Carotenoids

Carotenoids, the natural fat-soluble pigments are classified as carotenes (pure hydrocarbons with no oxygen) and xanthophylls (containing oxygen) that possess strong antioxidant activity [32]. The major phytochemicals that come under xanthophylls are identified to be crocetin and lutein. Similarly, the carotenes constitute lycopene, β-cryptoxanthin, α, β and γ-carotene. Among α, β and γ carotene, β-carotene plays an important role in human health. β-carotene is a red-orange pigment generally found in plants and fruits [32].

### 2.3 Glucosinolates

Glucosinolates (GLS), are a class of plant thioglucosides (organic compounds containing sulfur and nitrogen and are derived from glucose and amino acid) [33]. Isothiocyanates, indole and sulforaphane belong to this group. In the human digestive system, glucosinolates are hydrolyzed to isothiocyanates by the action of the enzyme myrosinase [34]. The enzymatic degradation helps the release of chemopreventive agents into the host system. The major dietary phyto-constituents indole in the acidic environment of the stomach is condensed and changed to a digestion derivative named di-indolylmethane (DIM) that exhibit strong anti-cancer property [34]. Sulforaphane is generally derived from cruciferous vegetables display effective anti-cancer potency in various cancer cell lines both in vivo and in vitro [34].

### 2.4 Organosulfur compounds (organosulphides)

Alliin, a sulfur-containing dietary phytochemical compound has a major derivative of the amino acid cysteine [35]. It has been reported to be the first natural carbon-sulfur-centered stereometric compound found in garlic species. Alliin is majorly found to have a potential role in immune response in the host organism [36]. Three additional sulfoxides (methiin, propiin and isoalliin) are also present in the tissues of onion [37].

### 2.5 Nitrogen-containing compounds

Among the plant-based secondary metabolites, the nitrogen-containing phytochemicals (alkaloids) have emerged as very prominent class of disease preventive antioxidants [38,39]. The chief dietary phytochemicals such as caffeine, codeine, noscapine and quinidine are placed in the major group of alkaloids [39]. Another group of nitrogen-containing phytochemicals; capsaicinoids are medically used as analgesics [40]. The major phyto-constituents of capsaicinoids include dihydrocapsaicin, homocapsaicin, capsaicin and nonivamide [40].

### 2.6 Terpenoids

All living organisms synthesize several terpenes for indispensable physiological functions. The classification of these natural phyto-products are based on the numeral of isoprenoid units present. The major classes of terpenoids consist of compounds belonging to monoterpenes, sesquiterpenes, diterpenes, sesterterpenes, triterpenes, and tetraterpenes. The dietary important terpenoids that have proven their potential as strong drug candidates include monotrepens (d-Limonene, d-carvone and perillyl alcohol) [41].

## 3 Diet, dietary phytochemicals and cancer

Diet plays a significant role in prevention of the cancer [42]. Though the interlink between diet and prevention of cancer has not yet been explicitly explained, there have been many pieces of evidence that suggest the importance of diet on limiting cancer risk. Studies have reported that cancers associated with the digestive tract were reduced almost half after daily consumption of dietary phytochemicals.

Consumption of polyphenol-rich fruits has been found to decrease the risk of cancer. Quercetin induces both apoptosis and autophagy in several tumor and cancer cell lines [43]. Anti-carcinogenic effects of resveratrol have efficiently reduced the skin and gastrointestinal tract tumor [7,8,11,44]. The chemo-preventive effect of curcumin isolated from turmeric is effective for the treatment of breast, lung and colon cancer, and brain tumor [45]. Epigallocatechin gallate (EGCG), the phyto-constituent present in green tea is favorable in treating prostate, bladder, cervical and brain cancers [42,[46], [47], [48]]. Similarly, Kaempferol is associated with reduction of pancreatic and lung cancer [49]. Gingerol, another active phyto-constituents isolated from fresh ginger is effective against tumors in colon, breast, ovary and pancreas origin [50].

Anthocyanidin, play an important role in reducing inflammation, thus limits tumor growth. Perillyl alcohol and d-limonene have displayed chemo-preventive activity against skin, liver, mammary and lung cancers. Owing to the effective antioxidant activity exhibited by β-carotene, it exhibits anti-cancer activity in several cancer cells and tumor cell lines [32]. The immunomodulatory function of β-carotene exhibits possible inhibition of carcinogenetic progress. In combination with vitamins E and C, β-carotene exhibits enhanced anti-cancer efficacy in several cancer cells. Lycopene exhibited potent anti-cancer effects against skin, breast, prostate, esophagus, stomach and colon cancers [32,[51], [52], [53]]. Glucosinolate derived from cruciferous vegetables has displayed chemo-preventive effect on liver, colon, mammary gland, lung, colorectal and pancreas cancer [54]. Di-indolylmethane have demonstrated exceptional anti-cancer effect against hormone-responsive breast, ovarian and prostate cancers (Table 2).

**Table 2:** Mechanism of chemoprevention by dietary phytochemicals in different cancer subtypes.

Sl. No	Cancer Subtype	Cell line	Compound name	Functional Involvement	Reference
1.	Breast and ovarian cancer	MCF-7	Fisetin	Mitochondrial apoptosis and autophagic cell death which is independent of apoptosis	[55]
2.		MDA-MB-231	Apigenin	Induction of autophagy via enhanced LC3 lipidation	[56]
3.		MCF-7	Genistein	Bax/Bcl-2 modulation for subsequent onset of apoptosis	[57]
4.		A2780	Genistein	Modulation the AKT signaling pathway for induction of autophagic cell death. Sustained onset of autophagy through modulation of PKC and ERK	[58]
5.		MDA-MB-231 and MCF-7	Kaempferol	Inhibition of cellular proliferation via G2/M phase cycle arrest. Induction DNA fragmentation, caspase 3, 7 and 9, Bax, PARP, and p53 for onset of apoptotic cell death.	[59]
6.		MDA-MB-361 and MCF-7	Epigallocatechin	ROS dependent onset of autophagy and apoptosis. Subsequent removal of dysfunctional mitochondria through mitophagy that mediate cell death	[47]
7.		MCF-7	Resveratrol	Autophagy independent of Beclin1 and Vps34 signalling pathway	Li et al., 200 9
8.		MCF-7	Codeine	Induction of apoptosis	[60]
9.	Cervical and prostate cancer	HeLa	Fisetin	Modulation of ERK1/2 and caspase-8 and caspase-3	[61]
10.		PC-3 and DU-145	Fisetin	Autophagic cell death though modulation of mTOR-AKT signaling pathway	[55]
11.		LNCaP	Genestein	Inhibition of invasion, migration and EMT	[58]
12.		PC-3 and DU145	Genestein	Inhibition of AKT/mTOR/p70S6K leading to autophagic cell death	[62]
13.		PC-3	β-carotene	Enhanced expression of cytochrome C and induction of caspase proteins	[63]
14.	Colon and gastric cancer	HCT-116	Apigenin	Autophagic cell death via inhibition of the PI3K/Akt/mTOR signalling pathway	[64]
15.		HT-29 and HCT-116	Kaempferol	Induction of TRAIL mediated apoptosis	[65]
16.		MKN-28, SGC-7901 and BGC-823 cells	Curcumin	Induction of both autophagic and apoptotic cell death	[45]
17.		HCT-116	Curcumin	Induction of autophagy associated senescence through enhanced LC3 lipidation	[66]
18.		SGC-7901 and MGC-803	Caffeine	Induction of caspase-3 and -9 mediated apoptosis	[67]
19.	Liver and pancreatic cancer	MIA PaCa-2	Genistein	Inhibition of the Bcl-2 expression for onset of apoptosis	[68]
20.		Hep3B	Andrographolide	Induction of apoptosis through modulation of MAPK, pJNK, ERK1/2 signalling	[69]
21.		HepG2	Oridonin	ROS mediated apoptosis for induction of apoptotic cell death through p53 and p38 expression, enhanced expression of caspase 3 and caspase-9	[70]
22.		HepG2	Cucurbitacin B	Regulation of the Bcl-2 expression, regulation of the cyclin D1 and cdc2 expression	[71]
23.	Glioma, melanoma and sarcoma	U87-MG and U373-MG	Curcumin	Induction of G2/M phase cycle arrest and AKT/mTOR/p70S6K mediated autophagy	[14]
24.		A375 and C8161	Curcumin	Autophagic cell death through regulation of AKT/mTOR/p70S6K signaling	[72]
25.	Head and neck carcinoma	Cal33 and FaDu	Gallic acid	Induction of apoptosis through upregulation of Bax, caspase-3 and downregulation of Bcl-2, NRF2, NQO1 and GCLC. Autophagic flux inhibition, enhanced LC3 lipidation.	[73,74]
26.	Lung cancer	A549	Curcumin	Regulation of AMPK, MAPK and ERK1/2 signalling	[45]
27.		A549 and H1299	Allicin	Inhibition of the cellular proliferation, invasion and metastasis via modulation of PI3K/AKT signaling	[75]
28.	Leukaemia	K562	Curcumin	Induction of autophagy via enhanced expression of Beclin1 and downregulation of Bcl-2, inhibition of AKT/mTOR/p70S6K	[76]
29.		CML	Resveratrol	Induction of autophagy through AMPK-mTOR signaling pathway	[14]
30.		CML	Quercetin	Induction of PKC mediated apoptosis	[77]
31.		HL-60	Codeine	DNA damage and nuclear fragmentation, caspase 3 activation for induction of apoptosis	[78]

Indoles and sulforaphane have been found to be more effective against breast cancer. Moreover, they have been influential in the protection of the skin against UV radiation damage. Sulforaphane has displayed very significant inhibitory effect on prostate tumorigenesis. Phenethyl isothiocyanate (PEITC) has been intensively studied for its chemo-preventive action against breast, lung, cervical, prostate and several myeloma cell lines. Effectively, PEITC has displayed very potent inhibitory activity against melanoma.

## 4 Chemo-preventive action of dietary phytochemicals

Carcinogenesis is documented as a complex, multistep process initiated with exposure to a carcinogenic agent [42]. In the cancer initiation stage, a normal cell gets transformed into a cancer cell. The cancer-initiating cell begins abnormal multiplication and gives rise to a heterozygous tumor cell population. In the tumor promotion stage, actively proliferating pre-neoplastic cells accumulate that lead to tumor progression which involves the growth of a tumor with potential invasion and metastatic potential. Chemoprevention is defined as the application of chemotherapeutic agents to inhibit, converse or hinder tumorigenesis/ carcinogenesis. Numerous dietary phytochemicals have been reported to act as chemo-preventive agents by interfering with a specific regulatory stage during the process of carcinogenic [79]. Overproduction of oxidants cause an oxidative imbalance that contributes towards oxidative damage of DNA, proteins and lipids to aid cancer pathophysiology [80]. In this context, to prevent oxidative stress dietary phytochemicals with potential antioxidant properties hold high importance as chemo-preventive agents [81].

Several phytochemicals seem to possess anti-inflammatory properties. The colossal interlink between cancer and inflammation is evident as several inflammatory conditions that influence the onset of cancer initiation [6,11,82,83]. Previous investigations have demonstrated that biological activity of these dietary phytochemicals are due to their potent free radicals scavenging and enhanced antioxidant activity that subsequently regulate the expression of oncogenes and tumor suppressor genes [5,6,10,11]. In addition to this, dietary phytochemicals regulate the cancer cell proliferation and differentiation, the arrest of cell cycle at different phases and onset of cell death pathways such as apoptosis and autophagy [11,84,85]. According to Lee Wattenberg’s conventional classification, the chemo-preventive agents are segmented into blocking and suppressing agents [86].

### 4.1 Blocking agents

As blocking agents, dietary phytochemicals prevent carcinogens to reach the target sites and subsequently inhibit the DNA damage [87]. These dietary phytochemicals neutralize the carcinogens by moderating the enzymatic systems responsible for them. These phyto-products either reduce their carcinogenic potential or increase their excretion [88,89]. Allyl sulfides present in garlic act as blocking agents by altering the host’s defense system against molecules that are responsible for DNA-damage. Similarly, tea polyphenols inhibit the binding of carcinogenic substances to genetic material preventing genetic mutations [90,91]. Quercetin, another prominent polyphenol increases the excretion of oxidative metabolites. Carotenoids react with the free radicals in a lipid-soluble environment to prevent oxidative stress. β-carotene supposed to inhibit cancer cell growth by enhancing cellular antioxidant propensity as well as improving immune response [92].

Kaempferol inhibits cellular proliferation through the induction of G2/M phase cycle arrest [59]. Moreover, it also regulates the expression of E-cadherin, N-cadherin, Slug and Snail for inhibition of EMT [59]. Genistein displays inhibition of cellular proliferation by arresting the cell cycle at the G2/M phase [93]. Moreover, inhibition of telomerase activity and angiogenic capacity support genistein-mediated chemo-prevention [58]. Moreover, apigenin-mediated inhibition of cellular proliferation is evident with G2/M phase cell cycle arrest [56]. Glucosinolates inhibit the enzyme activation to modify the steroid hormone metabolism and protect cells against oxidative damage, thus preventing tumor initiation [33]. They also expedite the cleansing of carcinogens by inducing Phase I and Phase II enzymes. Another antioxidant perillyl alcohol, protect cells from becoming cancerous, slow cancer cell growth and strengthen immune function to fight against cancer [33].

Allicin, another organic allyl sulfur compound exhibits inhibition of MMP-2 and MMP-9 expression for inhibition of cell proliferation [43]. It also regulates the STAT3 signalling pathway for inhibition of cell proliferation, migration and EMT [94]. Noscapine inhibits the cellular proliferation via binding to the tubulin microfilaments. In combination with docetaxel, noscapine binds to the tubulin for inhibition of cancer cell progression [95]. Noscapine-loaded nanocarriers in combination with doxorubicin enhanced the anti-cancer efficacy in several cancer cells [96]. Sulforaphane exhibits anti-cancer efficacy in several cancer cells through regulation of cell cycle progression, induction of apoptosis and autophagy, inhibition of angiogenesis and enhanced chemotherapeutic efficacy in combination with known anti-cancer drugs [63]. In colon and ovarian cancer cells, sulforaphane regulates the expression of HIF-1α and VEGF for inhibition of angiogenesis [97]. Gnidimacrin inhibits tumor progression through upregulation of p21WAF1/CIP1 signalling pathway [98]. In addition to this, gnidimacrin arrests the cell cycle at G2-phase and downregulates the expression of cdc2 [99].

Lycopene activates the phase II detoxification enzymes to reduce oxidative damage associated with cancer initiation [100,101]. Lycopene upregulates the cytochrome P450 expression to prevent carcinogenesis [102,103]. In several cancer cells, lycopene arrests the cell cycle at G0/G1 and S-phase [102,103]. Moreover, lycopene-mediated regulation of expression of MMP-2 and MMP-9 in several cancer cell lines inhibits the cancer growth and proliferation [[51], [52], [53]]. Lycopene exhibits anti-cancer efficacy through enhanced antioxidant, lipid peroxidation and ROS scavenging efficacy [32].

### 4.2 Suppressing agents

On the other hand, a certain class of dietary phytochemicals obstructs the malignant transformation of cancer-initiating cells by acting unswervingly on tumor cells and also by modifying their microenvironment through deploying hostile physiologic environments that are unfavorable for tumor growth and progression [104]. This group of phytochemicals inhibit tumor growth by induction of cell death pathways such as apoptosis [105]. In apoptosis deficient cancer cells, these phytochemicals deploy an alternative form of cell death known as autophagy [105]. Moreover, these dietary phytochemicals inhibit tumor angiogenesis [[106], [107], [108]]. Phenethyl isothiocyanate, curcumin and resveratrol have shown strong apoptosis and autophagy-inducing potential in several cancer cell lines [109]. These phytochemicals are known to regulate the pro-apoptotic and anti-apoptotic proteins that are responsible for the onset of apoptosis [110]. Moreover, they also regulate autophagy and autophagy-associated genes to regulate autophagic cell death [110].

Quercetin induces autophagy through induction of MAPK/PI3K/PKC/ERK signalling pathway [111,112]. Sulforaphane induces chemo-preventive action through the regulation of the NRF2 signaling pathway in several cancer cell lines [113,114]. Moreover, sulforaphane also regulates several epigenetic regulatory mechanisms for cancer prevention. Mechanistically, sulforaphane reverses the epigenetic alteration via DNA methyltransferases and histone deacetyltransferases and several non-coding RNAs [113]. Sulforaphane also alters the apoptotic signal through upregulation of Bax and downregulation of Bcl-2 expression [115]. Allicin activates the caspase cascades for induction of apoptosis. In addition to this, allicin upregulates the Bax and downregulates the Bcl-2 expression for induction of apoptosis [94]. Noscapine induces ROS for subsequent activation of apoptosis [116]. In several tumor cells, prolonged exposure to apigenin inhibits autophagy through inhibition of Beclin-1 expression that subsequently promotes caspase 3 and 9 dependent apoptosis [56]. In addition to this, apigenin induces apoptosis through downregulation of Bcl-2 and upregulation of caspase 3 expressions. Kaempferol mediated inhibition of PI3K/AKT/mTOR signaling, enhanced expression of Beclin-1, ATG-5, ATG-7, ATG-12 genes and enhanced LC3 lipidation for induction of autophagic cell death [117].

## 5 Chemotherapeutic efficacy of dietary phytochemicals in different cancer subtypes

### 5.1 Breast and ovarian cancer

**Fig. 3:**
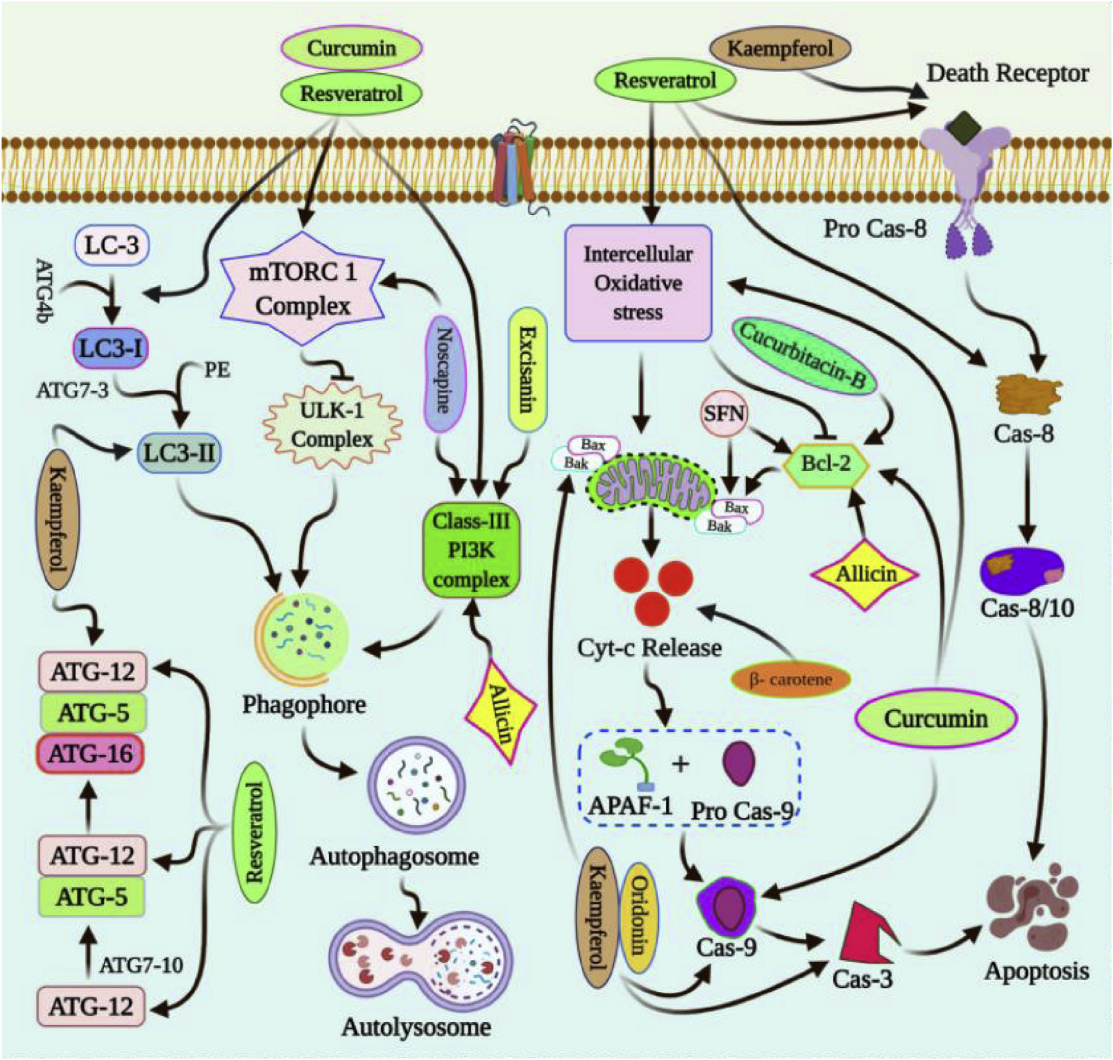
Molecular mechanisms of chemoprevention by dietary phytochemicals. Polyphenols like curcumin, resveratrol and noscapine regulate autophagic cell death via inhibition of mTORC1 signalling. Similarly, curcumin, resveratrol, noscapine, allicin, and excisanin activate the PI3K signalling for elongation of extending phagophore. Kaempferol, curcumin and resveratrol enhance the LC3 lipidation in the extending autophagosomes. Resveratrol enhances the expression of ATG-5 and ATG-12. Similarly, kaempferol and resveratrol also regulate the ATG-16 formation from ATG-5 and ATG-12. Curcumin, resveratrol and kaempferol enhance the Bax/Bcl-2 expression to regulate apoptosis. In addition to this, kaempferol, oridonin and curcumin upregulate the caspase cascades responsible for the onset of apoptosis. Oridonin regulates the expression of caspase 3 and caspase 9 while cucurbitacin B and allicin regulate the Bcl-2 expression for induction of apoptotic cell death. Sulforaphane also alters the apoptotic signal through upregulation of Bax and downregulation of Bcl-2. β-carotene enhances the release of cytochrome C to mediate mitochondrial apoptosis. Resveratrol enhances the expression of caspase 8 during the onset of extrinsic apoptosis while kaempferol elicits the death receptors that are responsible for extrinsic apoptosis.

In MCF-7 breast cancer cells, fisetin induces intrinsic apoptosis and autophagic cell death which is independent of apoptosis [55]. In MDA-MB-231 cells, apigenin induces autophagy via enhanced LC3 lipidation [56,64,118,119]. In MCF-7 cells, genistein enhances the Bax/Bcl-2 expression for subsequent onset of apoptosis [57]. In A2780 cells, genistein upregulates the AKT signaling pathway for induction of autophagic cell death. Moreover, genistein sustains the onset of autophagy through upregulation of PKC and ERK signaling [58]. In MDA-MB-231 and MCF-7 cells, kaempferol inhibits cellular proliferation via G2/M phase cell cycle arrest. Furthermore, it induces DNA fragmentation for the induction of apoptotic cell death [59]. Mechanistically, kaempferol regulates the expression of caspase 3, 7 and 9, Bax, PARP and p53 to induce apoptosis [59]. In addition to this, SOD and CAT regulation by kaempferol regulates the onset of apoptosis [59]. In MCF-7 and MDA-MB-361, EGCG induces ROS dependent onset of autophagy and apoptosis [47,48]. The mitochondrial dysfunction upon treatment of EGCG and their subsequent removal through mitophagy aid EGCG mediated autophagic cell death [47,48]. In the breast cancer-bearing xenograft mice model, EGCG inhibits lung metastasis by inducing apoptosis through DNA damage [47,120,121]. In MCF-7 cells, resveratrol induces autophagy independent of Beclin-1 and Vps34 signalling pathway [[122], [123], [124]]. In ovarian cancer cell lines, resveratrol induces autophagy through inhibition of PI3K/AKT/mTOR signalling pathway. In ovarian cancer cells, sulforaphane regulates the expression of HIF-1α and VEGF expression for inhibition of angiogenesis [97]. In MCF-7 cells, codeine induces apoptosis in a dose-dependent manner [60] (Fig. 3, Table 2).

### 5.2 Cervical and prostate cancer

Fisetin, a naturally occurring flavonoid induces apoptosis in the human cervical cancer cell line (HeLa) through upregulation of ERK1/2 and caspase 8 and caspase 3 [61]. In HeLa cells, quercetin induces autophagy and apoptosis for subsequent inhibition of cancer cell survival and proliferation [125,126]. In PC-3 and DU-145 cells, fisetin induces autophagic cell death through inhibition of mTOR-AKT signaling pathway [55]. In LNCaP cells, genestein treatment inhibits invasion, migration and EMT [58]. A similar regulation of inhibition of AKT/mTOR/p70S6K mechanistic involvement of signaling pathways is also responsible for the induction of autophagic cell death in PC-3 and DU-145 cells [62]. In PC-3 cells, β-carotene exhibits apoptosis through enhanced expression of cytochrome C and induction of caspase proteins. Sulforaphane has displayed very significant inhibitory effects on prostate tumorigenesis [63]. In human prostate cancer cells, paclitaxel in combination with noscapine exhibits enhanced anti-cancer activity [127,128] (Fig. 3).

### 5.3 Colon and gastric cancer

Apigenin induces autophagic cell death in human colon cancer (HCT-116) cells via inhibition of the PI3K/AKT/mTOR signalling pathway [56,64,118,119]. In HT-29 and HCT-116 cells, kaempferol induces TRAIL-mediated apoptosis [65]. In HT-29 and SSC-4 cells, EGCG elicits the onset of apoptosis for suppression of cell cellular proliferation and induction of cell death [47]. In MKN-28, SGC-7901 and BGC-823 cells, curcumin induces both autophagic and apoptotic cell death for cancer inhibition [45]. In HCT-116 cells, curcumin induces autophagy-associated senescence through enhanced LC3 lipidation [66]. In HT-29 cells, resveratrol induces ROS-dependent apoptosis [129]. In colon cancer cells, sulforaphane downregulates the expression of HIF-1α and VEGF expression for inhibition of angiogenesis [97]. In SGC-7901 and MGC-803 cells, caffeine induces caspase 3 and 9 mediated apoptosis [67]. In AGS cells, codeine induces apoptosis in a dose-dependent manner [60]. In human colon cancer cells, noscapine induces apoptosis via enhanced PTEN/PI3K/mTOR signaling [130] (Fig. 3, Table 2).

### 5.4 Liver and pancreatic cancer

In HepG2 cells, kaempferol induces apoptosis via regulation of ROS [59]. In MIA PaCa-2 cells, genistein downregulates the Bcl-2 expression for the onset of apoptosis. In combination with 5-FU, genistein exerts apoptosis in human pancreatic cells [68]. Andrographolide inhibits the growth of Hep3B cells by induction of apoptosis through the upregulation of MAPK, pJNK, ERK1/2 signalling pathways [69]. Excisanin A also decreased the cellular viability of Hep3B cells via induction of apoptosis through downregulation of AKT signaling [131]. Oridonin promotes the ROS-mediated apoptosis for the induction of apoptotic cell death in HepG2 cells [70]. Moreover, it also regulates p53 and p38 expression [132]. Oridonin regulates the expression of caspase 3 and caspase 9 for induction of apoptosis. Cucurbitacin B regulates the Bcl-2 expression in HepG2 cells for induction of apoptosis [133]. Moreover, it also regulates the cyclin D1 and cdc2 expression for the suppression of cellular proliferation [71] (Fig. 3, Table 2).

### 5.5 Glioma, melanoma and sarcoma

Curcumin induces G2/M phase cycle arrest and AKT/mTOR/p70S6K mediated autophagy in U87-MG and U373-MG cells [14,134,135]. In A375 and C8161 cells, curcumin induces autophagic cell death through the regulation of AKT/mTOR/p70S6K signaling [72,136]. In MG63 cells, quercetin in synergism with pharmacological and genetic inhibitors of autophagy induces apoptosis for inhibition of cell proliferation [125,126]. Noscapine also exhibits anti-cancer efficacy against glioblastoma by inhibiting the microtubules [137]. Caffeine induces selective cytotoxicity and DNA damage in human sarcoma cell lines [138]. Moreover, synergistic treatment of caffeine and conventional chemotherapeutic drugs exhibits enhanced chemo-sensitization through induction of apoptosis. In human osteosarcoma, caffeine induces G0/G1 phase cell cycle arrest and caspase 3/7 activation for the induction of apoptosis [139] (Fig. 3, Table 2).

### 5.6 Head and neck carcinoma

In oral cancer cells, curcumin induces autophagic cell death through bulk cellular vacuolation and enhanced conversion of LC3I to LC3II [45]. Similarly, in Cal33 oral cancer cells, gallic acid induces apoptosis through upregulation of Bax, caspase 3 and downregulation of Bcl-2 [74]. Moreover, it inhibits autophagy through enhanced expression of p62 to induce autophagy associated cell death. Gallic acid in combination with gamma irradiation inhibits lipophagy to promote lipophagy associated cell death [73]. A synergistic combination of sulforaphane and paclitaxel in Barrett esophageal adenocarcinoma, an enhanced antiproliferation is evident through the onset of apoptosis [140]. Moreover, it inhibits bronchial dysplasia and cellular proliferation which is evident through reduced Ki-67 expression (Fig. 3, Table 2).

### 5.7 Lung cancer

In A549 cells, curcumin regulates the AMPK, MAPK and ERK1/2 signaling pathway associated with cellular transformation, differentiation and proliferation [45]. In smoking-associated lung cancer patients, sulforaphane induces apoptosis through enhancement of caspases expression [63]. In A549 and H1299 cells, allicin inhibits the cellular proliferation, invasion and metastasis via regulation of PI3K/AKT signaling [75] (Fig. 3, Table 2).

### 5.8 Leukaemia

In K562 cells, curcumin induces autophagy via enhanced expression of Beclin-1 and downregulation of Bcl-2 [76]. Moreover, the inhibition of AKT/mTOR/p70S6K signaling is also responsible for the onset of autophagy [76]. Similarly, in myeloma and leukemia cells, resveratrol induces the Fas and its associated ligands as well as Bcl-2 and Bax signaling for the onset of both extrinsic and intrinsic apoptosis [141,142]. In chronic myeloid leukemia, resveratrol induces autophagy through the AMPK-mTOR signaling pathway [14,111,134]. In chronic lymphocytic leukemia, quercetin induces PKC-mediated apoptosis [77,143,144]. In HL-60 cells, codeine induces DNA damage and nuclear fragmentation for induction of apoptosis [145]. In HL-60 and HSC-2 cells, codeine activates the caspase 3 mediated apoptosis [78] (Fig. 3, Table 2).

## 6 Preclinical efficacy of dietary phytochemicals

In breast cancer (MCF-7) bearing mice xenograft model, curcumin exhibited anti-tumor efficacy in combination with paclitaxel and chemo-sensitize the tumor cells towards apoptosis [146]. In another breast cancer (MDA-MB-245) bearing mice xenograft model, curcumin and paclitaxel inhibited the NF-κβ and MMP signalling for inhibition of tumor metastasis [135]. Resveratrol regulated the breast cancer stem cells via regulating the Wnt/ β-catenin in breast cancer-bearing mice xenograft model [42]. In nude mice bearing MDA-MB-231 cells exhibit reduced angiogenesis and enhanced apoptosis post-treatment with resveratrol [42,135]. In addition to this, resveratrol mediated PI3K/AKT/mTOR signalling inhibition induced autophagic cell death. EGCG induced DNA damage as the precursor of apoptosis in nude mice model bearing breast tumor [121]. In addition to this, it also reduced the invasiveness of breast tumor thereby suppressing the lung metastasis. Fisetin inhibited the in vivo breast tumor growth via induction of apoptosis in a caspase-dependent manner [61]. Sulforaphane suppressed the tumor growth of triple-negative breast cancer cells, via inhibition of the Cripto/Alk4 protein complex formation [147].

In prostate cancer-bearing mice model, curcumin inhibited the tumor growth and elicited induction of apoptosis via NF-κβ, MAPK and EGFR regulation. In TRAMP rat model, resveratrol reduced the growth of prostate cancer tumor via downregulation of ERK1/2 signalling and induction of apoptosis [135]. In PC-3 bearing mice xenograft model, lycopene reduced the angiogenesis via inhibition of VEGF and EGF expression [148]. In PC-3 cells bearing prostate cancer mice model, sulforaphane retarded the tumor growth via induction of apoptosis in a caspase-dependent manner [149]. In xenograft mice model bearing colorectal cancer, curcumin treatment sensitizes the tumor cells towards chemotherapy via inhibition of NF-κβ signalling. Curcumin in synergism with oxaliplatin or 5-fluorouracil inhibited tumor growth in human gastric cancer-bearing mice model [135]. Lycopene inhibited the growth of colorectal cancer via decreasing the PCNA, COX-2, MMP-9, ERK1/2 and induction of apoptosis via upregulation of caspase 3 and p21 [148]. In a xenograft tumor model in mice, noscapine induced apoptosis to restrain tumor growth via downregulation of Bcl-2 and upregulation of Bax, cytochrome C, caspase 3 and caspase 9 [150].

Curcumin in synergism with metformin decreased hepatocellular carcinoma tumor growth in xenograft mice model [151]. In SK-Hep-1 hepatoma cells bearing mice model, lycopene inhibited the tumor growth and metastasis via decreased expression of MMP-2 and MMP-9 [148]. In the human glioma cell bearing mice model, curcumin mediated inhibition of angiogenesis was by decreased MMP-9 expression [152]. In the A375 cell bearing melanoma mice model, curcumin inhibited the PI3K/AKT/mTOR signalling to induced autophagic cell death [153]. In DMBA induced skin tumor-bearing CD-1 mice model, resveratrol inhibited the tumor growth via downregulation of TGF-β and TNF-α and other inflammation-related key molecular proteins [154]. Apigenin induced apoptosis for reduction of chondrosarcoma tumor in mice model. Resveratrol reduced the tumor growth, angiogenesis and metastasis in Lewis Lung Carcinoma bearing C57BI/6 mice model [155]. Apigenin inhibited the tumor growth, metastasis and angiogenesis in the NSCLS xenograft mice model [42]. In the murine xenograft lung cancer model, noscapine in synergism with cisplatin reduced the tumor growth via induction of apoptosis with evident upregulation of caspase 3, caspase 8, PARP, p53, p21 and Bax [156].

## 7 Challenges in therapeutic intervention and clinical translation

The limited bioavailability, poor stability and permeability, reduced pharmacokinetics (reduced plasma, blood and tissue concentration) and pharmacodynamics, metabolism and absorption has been the major challenges for clinical translation of dietary phytochemicals. Then extensive digestion of curcumin is a major obstacle for maintaining higher plasma and tissue concentration [157]. In a phase II clinical trial (NCT00094445), even after 8 weeks of application of curcumin, the plasma concentration was found to be very low. Curcumin at 2.5−5 μg/mL concentration induced chromosomal alteration and DNA damage [158]. Curcumin at doses of 0.9–3.6 g/day for 14 days exhibited nausea and diarrhea [159]. Oleoresin has shown toxic and carcinogenic effect in in vivo rat model [157]. SRT501 in combination with bortezomib approved for multiple myeloma has noticed adverse renal toxicity, nausea, diarrhea, vomiting, fatigue and anemia [160,161]. In Male Sprague-Dawley rats, low bioavailability of kaemferol (nearly 2%) has reduced its use as chemopreventive in several cancer subtypes [162]. Similarly, the poor solubility, limited bioavailability, instability and poor permeability have also limited the use of quercetin as potent chemopreventive [163].

## 8 Conclusion and future perspective

Ever-increasing cancer incidences and associated mortalities despite advancement in prognosis, diagnosis and treatment have emerged as foremost challenge in cancer therapy. The cytotoxicity and non-target specificity have contributed to more problems with the application of chemotherapeutic drugs. To abide by the ill effects associated with chemotherapy, dietary phytochemicals have emerged as potent pharmacophores in cancer therapeutics. With mechanistic involvement of cell survival and cell death pathways (apoptosis and autophagy) associated with cancer-treating drugs, identification and evaluation of dietary phytochemical modulating such signaling pathways has appeared as possible pharmacophores for future generation cancer treatment. With apoptotic and autophagic target specificity, dietary phytochemicals will uncover several associated cellular networking that are potential targets for cancer chemoprevention. Moreover, as individual drug candidates and in combination with conventional cancer drugs, these phytochemicals will enhance chemo-sensitization for better chemotherapy. In addition to this, identifying and understanding the molecular key players involved, target-specific drug delivery through nano carriers, liposomes, polymers and micro emulsions will be formulated for enhanced bioactivity and bioavailability. Moreover, the identification of such key molecules will uncover novel targets for future generation personalized and precision medicine in cancer therapy.

## Author contributions

SP, RN, SMP, BP. BS and CB have prepared the manuscript. MJ and SKB have done the conceptualization and proofreading of the manuscript.

## Research involving human participants and/or animals

No Human participation and/or Animal have been used in this study

## Informed consent

The corresponding author on behalf of all coauthors agree to accept the informed consent of compliance with ethical standard

## Declaration of Competing Interest

The authors have no conflicts of interest to disclose.
